# Urological symptoms following ketamine treatment for psychiatric disorders: A systematic review

**DOI:** 10.1177/02698811251350267

**Published:** 2025-06-30

**Authors:** Jess Kerr-Gaffney, Anna Tröger, Alice Caulfield, Philipp Ritter, James Rucker, Allan H Young

**Affiliations:** 1 Department of Psychological Medicine, Centre for Affective Disorders, Institute of Psychiatry, Psychology and Neuroscience, King’s College London, London, UK; 2 Department of Psychiatry and Psychotherapy, University Hospital Carl Gustav Carus at the TU-Dresden, Dresden, Germany

**Keywords:** Ketamine, depression, adverse event, bladder, urology

## Abstract

Ketamine has emerged as a putative rapid-acting treatment option for psychiatric disorders, particularly treatment-resistant depression. Chronic recreational ketamine use is associated with ketamine-induced urological toxicity, raising concerns over the safety of repeated ketamine treatments. This systematic review aimed to synthesise urological findings from clinical trials and observational studies using ketamine for the treatment of psychiatric disorders. Electronic databases were searched up until 4th April 2024 for trials and observational studies using ketamine treatment for psychiatric disorders in adults, and which reported assessment of urinary, bladder or renal symptoms. Twenty-seven studies were included, mostly in depressive disorders (*N* = 24). Urological symptoms were reported in 0%–24.5% of patients receiving ketamine treatment; symptoms tended to be mild or moderate in severity. Where reported, continuous outcome measures (urinary parameters and symptom questionnaires) did not show significant changes from baseline to follow-up. Only 15% of studies were rated low risk of bias. Most studies did not assess long-term ketamine treatment, and many included undefined or passive monitoring of urological symptoms rather than systematic assessment. Based on the limited data available, ketamine treatment does not appear to be associated with elevated risk of urological symptoms; however, further long-term studies are required.

## Introduction

Ketamine was synthesised in 1962 as an alternative to phencyclidine, offering a shorter duration of action and reduced risk of delirium (Domino, 1980). It has been used as an anaesthetic globally for over 50 years. Concurrently, its potential applications in psychiatry were explored, with early studies using subanaesthetic doses (0.5–1.0 mg/kg) alongside psychotherapy to treat a range of illnesses such as depression, anxiety and alcohol use disorder (Khorramzadeh and Lotfy, 1973; Krupitsky and Grinenko, 1997). In the first randomised controlled trial (RCT), Berman et al. (2000) demonstrated significant improvement in depressive symptoms within 24 h and lasting for approximately 72 h of ketamine infusion compared to placebo in patients with unipolar or bipolar depression. Since then, ketamine’s antidepressant effects have been demonstrated in phase III trials (Loo et al., 2023; Reif et al., 2023), and evidence is accumulating for its use in alcohol use disorder (Grabski et al., 2022). Small open-label studies have also suggested potential for treating post-traumatic stress disorder (PTSD; Albott et al., 2018), anorexia nervosa (Mills et al., 1998) and obsessive-compulsive disorder (Bloch et al., 2012). Whilst intravenous (IV) administration has been used most extensively, subcutaneous, oral, intranasal and intramuscular routes have also been explored.

Ketamine is a non-competitive N-methyl-D-aspartate (NMDA) receptor antagonist, likely contributing to both its antidepressant and anaesthetic effects. However, ketamine has a complex pharmacology, additionally interacting with serotonin, dopamine, opioid, gamma-aminobutyric acid (GABA) and cholinergic systems (Zanos et al., 2018). (R,S)-ketamine, a racemic mixture of the two enantiomers (R)-ketamine (arketamine) and (S)-ketamine (esketamine), is most widely used in medicine. (S)-ketamine is more potent than (R)-ketamine due to a three- to four-fold higher affinity for the NMDA receptor (Ebert et al., 1997), and therefore was developed commercially as a novel antidepressant. Phase II and III trials demonstrated efficacy of intranasal (S)-ketamine plus an oral antidepressant over placebo (Daly et al., 2019; Popova et al., 2019), leading to US Food and Drug Administration (FDA) and European Medicines Agency approval of Spravato^TM^ nasal spray for treatment resistant depression (TRD). Despite this, (S)-ketamine is more effective than racemic ketamine, with meta-analyses showing similar or smaller antidepressant effects with (S)-ketamine compared to racemic ketamine (Bahji et al., 2021; Dean et al., 2021). Whilst early studies suggested that (R)-ketamine might have its own independent more potent and longer-lasting antidepressant effects than (S)-ketamine alongside fewer side effects (Hashimoto, 2020), subsequent RCTs found no significant antidepressant effects when compared to placebo (Leal et al., 2023; atai Life Sciences, 2023).

Acute adverse events reported with ketamine treatment include sedation, elevated blood pressure, dizziness, dissociation, nausea and headaches, most of which resolve on the same dosing day (Short et al., 2018). Longer-term side effects of ketamine treatment are not well characterised; however, some have raised concerns over the potential for addiction given widespread and increasing use of ketamine as a recreational drug, possibly facilitated by ketamine’s dopaminergic effects (Morgan and Curran, 2012). However, long-term trials of ketamine treatment have not documented cases of abuse following treatment for TRD (Wajs et al., 2020). Frequent recreational use of ketamine has also been associated with impairments in short- and long-term memory (Morgan and Curran, 2006); however, again, these have not borne out in trials of ketamine treatment (Morrison et al., 2024; Wajs et al., 2020). Other acute risks associated with recreational use are accidental trauma, abdominal pain, tachycardia and adverse psychological experiences (Weiner et al., 2000).

Ketamine-induced urological toxicity (KIUT) is associated with chronic recreational use of ketamine, with around one-quarter of ketamine users reporting lower urinary tract symptoms (Winstock et al., 2012). Symptoms include increased urinary frequency and urgency, haematuria, dysuria, nocturia and lower abdominal pain. Multiple mechanisms likely contribute to KIUT, including bladder inflammation, urothelial barrier damage, direct toxicity of ketamine in the urine, nerve hyperplasia and hypersensitivity, cell apoptosis and microvascular damage (Ng et al., 2021). Contraction of the bladder, increased thickness of the bladder wall, urothelial denudation, and the presence of eosinophils and mast cells in the urothelium are common pathological findings, and chronic kidney failure can result in severe cases (Anderson et al., 2022). In most cases of early KIUT, cessation of ketamine use will result in recovery of the urinary tract and symptom improvement. Pharmacological management can include non-steroidal anti-inflammatory drugs and anticholinergics, and where these are unsuccessful, hyaluronic acid solution and botulinum toxin-A injections are also treatment options (Anderson et al., 2022). In later stages, surgical interventions such as ureteral implantation or augmentation enterocystoplasty may be required. Due to the substantial pain associated with KIUT, the analgesic effect of ketamine may, in fact, reinforce use (Wood et al., 2011).

Although risk of urological complications is related to frequency and dose, where higher doses (⩾1 g/session) and more frequent use (⩾9 days/month) are associated with higher rates of symptoms (Winstock et al., 2012), urinary symptoms are also reported in a small proportion of less frequent users (>2 days/month; Muetzelfeldt et al., 2008). Severe urological symptoms have also been documented in patients prescribed ketamine for pain (Grégoire et al., 2008; Storr and Quibell, 2009). Since the antidepressant effects of a single dose of ketamine are relatively short-lived (Romeo et al., 2015), treatment protocols for IV ketamine generally involve an acute phase of treatment (e.g. 2–6 weeks, 1–3 doses per week) followed by a maintenance phase, with weekly, fortnightly or monthly doses (Andrade, 2017). In treatment-refractory cases, maintenance treatment may last years. With this in mind, possible urological side effects of ketamine treatment should be monitored. However, a previous review of ketamine treatment for depression found that most adverse event monitoring was only conducted during the acute exposure period (a few hours; Short et al., 2018); therefore, little is known about urological effects during repeat-dose ketamine treatment. The aim of this systematic review was therefore to synthesise urological findings from clinical trials and observational studies using ketamine for the treatment of psychiatric disorders.

## Methods

### Systematic review protocol

This systematic review and meta-analysis were reported in accordance with the Preferred Reporting Items for Systematic Reviews and Meta-Analyses (PRISMA) guidelines (Page et al., 2021). The protocol was preregistered on PROSPERO (ID: CRD42024529999). There was one change to the original protocol: studies including patients with schizoaffective disorder where ketamine was used to treat depressive episodes were included, given that ketamine is sometimes used off-label in this population (Aziz et al., 2017).

### Information sources and search strategy

Three electronic databases (PubMed, Web of Science and PsycInfo) were searched for papers up until 4th April 2024. There was no lower search date limit. Searches were performed using the following terms: Ketamine OR esketamine OR arketamine OR S-ketamine OR R-ketamine AND ‘Major depressive disorder’ OR MDD OR Depression OR bipolar OR mania OR cyclothymi* OR ‘TRD’ OR ‘treatment resistant depression’ OR anxiety OR phobia OR panic OR agoraphobia OR OCD OR ‘obsessive compulsive disorder’ OR ‘post-traumatic stress disorder’ OR PTSD OR ‘eating disorder’ OR ‘anorexia nervosa’ OR ‘bulimia nervosa’ OR ‘binge eating disorder’ OR ‘avoidant restrictive food intake disorder’ OR suicide* OR ‘alcohol use disorder’ OR ‘substance dependence’ OR addiction OR ‘substance use disorder’ OR ‘cannabis use disorder’ OR ‘opioid use disorder’ OR ‘stimulant use disorder’ OR ‘cocaine use disorder’ OR ‘tobacco use disorder’ OR cigarette OR ‘gambling disorder’ OR ‘sleep disorder’ OR insomnia OR ‘personality disorder’ AND Cystitis OR urolog* OR urinary OR bladder OR ureter OR kidney OR renal OR polyuria OR nocturia OR dysuria OR urine OR haematuria OR proteinuria OR ‘bladder pain’. No search limits were applied. Reference lists of relevant review articles and eligible papers were also searched.

### Inclusion and exclusion criteria

Inclusion criteria for studies were as follows: (a) RCTs, single-arm trials, open-label trials, and observational studies, (b) including adults ⩾18 with a diagnosis of a psychiatric disorder, including substance use disorders, (c) subanaesthetic doses of racemic ketamine, S-ketamine or R-ketamine, were administered via IV, intranasal, oral, subcutaneous, intramuscular or sublingual routes, for treatment of a psychiatric disorder, (d) active or inactive placebo, or no comparator, (e) reports assessment of and data for any urinary, bladder, or renal symptoms or outcome measures and (f) full text in English or German published in a peer-reviewed journal. Exclusion criteria were (a) case studies, commentaries and reviews; (b) neurodevelopmental, neurodegenerative, psychotic (with the exception of depressive episodes in schizoaffective disorder) or dissociative disorders and (c) ketamine treatment was used for general health conditions or as an analgesic/anaesthetic.

### Study selection

After all records were retrieved and duplicates removed, titles and abstracts were independently screened by two authors (JKG and AT) using Covidence software. Where titles and abstracts appeared relevant, these were retained, and full texts were retrieved. Full-text articles were assessed for eligibility independently by the same two authors, and any discrepancies were discussed until a resolution was reached.

Where urological symptoms were assessed but data were not reported, the study authors were contacted for data, and the paper included this if this was provided.

### Data extraction

The following information was extracted from each article: number of participants, population, study design, intervention (dose, route, no. of administrations, treatment duration, details on comparator if any), gender and mean age of participants, concomitant medications or therapies, comorbidities, duration of follow-up of AE assessment, frequency of urinary symptoms or mean and variance in urinary parameters at all available time points.

### Risk of bias

Risk of bias was assessed independently by two authors (AT and AC) using Cochrane’s risk of bias tools: the RoB v2 for RCTs (Sterne et al., 2019) and the Risk of Bias In Non-randomised Studies of Interventions (ROBINS-I) for non-randomised studies (Sterne et al., 2016). A third author (JKG) resolved any discrepancies. Overall risk of bias ratings were calculated using the guidance specified by each tool.

### Synthesis of results

A narrative summary of results summarises findings across studies, grouped by population.

## Results

### Study selection

**Figure 1.**
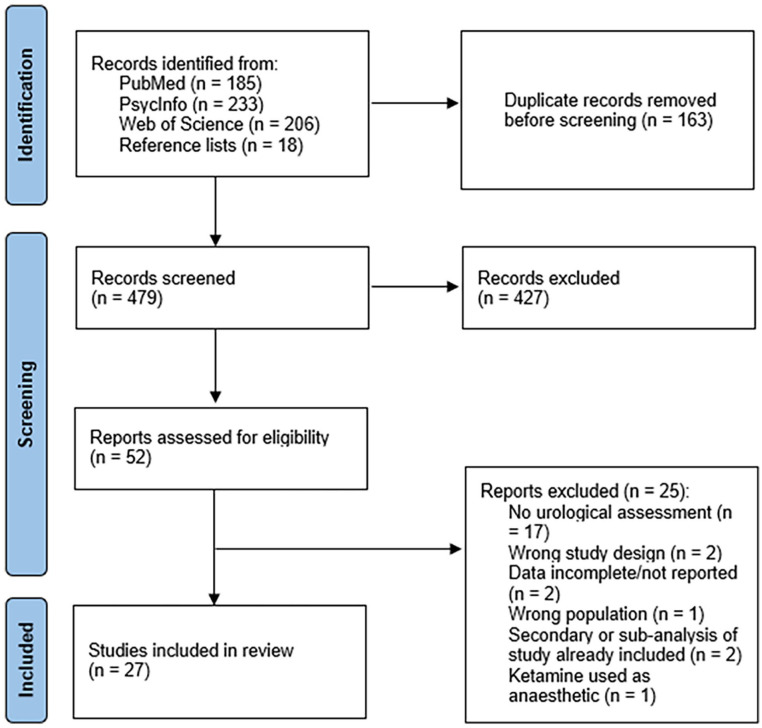
Systematic review search flow diagram.

The results of the study screening process are presented in Figure 1. Twenty-seven articles met all eligibility criteria and were included in the review.

### Study characteristics

Included studies are shown in Table 1. Most studies (*n* = 24) included patients with depressive disorders: unipolar or bipolar TRD (*n* = 20), mixed samples of major depressive disorder (MDD), bipolar disorder (BD) or schizoaffective disorder (*n* = 2), MDD only (*n* = 1) and MDD with chronic suicidality (*n* = 1). The remainder included patients with chronic PTSD (*n* = 2) or treatment-refractory general anxiety disorder (GAD) and/or social anxiety disorder (SAD; *n* = 1). Mean age ranged from 32.0 (Glue et al., 2018) to 70.6 years (Ochs-Ross et al., 2020). The proportion of female participants ranged from 0 (Gálvez et al., 2018) to 91% (Archer et al., 2018). Fifteen studies were RCTs, five of which included a crossover component, and three of which included an open-label phase either before or after the RCT phase. The remaining studies were retrospective cohort studies or chart reviews (*n* = 7) or one-arm open-label trials (*n* = 5).

**Table 1.** Study characteristics.

Study	Design	Population	Intervention					Follow-up	Analysis groups	Sex (% female)	Mean age (years)	Urinary symptom assessment	Assessment frequency
			Formulation	Route	Dose	Schedule	Treatment duration						
Al Shirawi et al. (2017)	Retrospective	TRD (MDD)	NR (assume racemic)	Oral	Mean 222 mg, max. 300 mg	3 doses/week	At least 4 weeks	NR	22 ketamine	59	39.0	AEs	NR
Archer et al. (2018)	Retrospective	TRD (MDD and BD)	NR (assume racemic)	IV	0.5 mg/kg	Acute: 2 doses/week, maintenance: variable	6–49 weeks	NR	11 ketamine	91	51.3	AEs	NR
Can et al. (2021)	OL one-arm	MDD + chronic suicidality	Racemic	Oral	Start: 0.5 mg/kg, max: 3.0 mg/kg	1 dose/week	6 weeks	6 weeks^a^	32 ketamine	53	45.7	PRISE	Weekly
Costi et al. (2019)	OL, then RCT^b^	TRD (MDD)	NR (assume racemic)	IV	0.5 mg/kg	2 doses/week	2 weeks	4 weeks^a^	18 ketamine + lithium	50	45.0	AEs	Twice weekly
									16 ketamine + placebo	56	45.8		
Daly et al. (2018)	RCT with crossover for placebo, then OL	TRD (MDD)	Esketamine	Intranasal	28, 56 or 84 mg	2 doses/week	2 weeks	2 weeks^a^	17 esketamine 84 mg	50	49.8	AEs	NR
									20 esketamine 56 mg	82	42.7		
									28 esketamine 28 mg	46	42.1		
									33 placebo	55	44.4		
Daly et al. (2019)	Withdrawal RCT	TRD (MDD)	Esketamine	Intranasal	56 or 84 mg	Acute: 2 doses/week, maintenance: 1 dose/week or fortnightly	At least 16 weeks	At least 18 weeks	90 remitters, esketamine + antidepressant	64	45.4	BPIC-SS, AEs	NR
									86 remitters, placebo + antidepressant	69	46.2		
									62 responders, esketamine + antidepressant	61	47.2		
									59 responders, placebo + antidepressant	71	46.7		
d’Andrea et al. (2024)	Retropective	TRD (MDD)	Esketamine	Intranasal	28, 56 or 84 mg	2 doses/week	4 weeks	4 weeks	140 esketamine nasal spray	54	49.4	AEs	Monthly or less. During infusion and at 1 follow-up point
			Racemic	IV	0.5 mg/kg, max. 0.75 mg/kg	2 doses/week	2 weeks	4 weeks	171 ketamine IV	50	47.5		
Fava et al. (2020)	RCT	TRD (MDD)	NR (assume racemic)	IV	0.1, 0.2, 0.5 or 1.0 mg/kg	1 dose	1 day	4 weeks	18 0.1 mg/kg ketamine	56	43.1	SAFTEE-S	Several times in the first week, then every 2 weeks
									20 0.2 mg/kg ketamine	45	45.5		
									22 0.5 mg/kg ketamine	50	48.6		
									20 1.0 mg/kg ketamine	40	47.4		
									19 midazolam	58	45.6		
Feder et al. (2014)	Crossover RCT	Chronic PTSD	NR (assume racemic)	IV	0.5 mg/kg	1 dose	2 weeks	1 day^a^	22 ketamine^c^	59	36.4	PRISE	During infusion and at 1 follow-up point
									19 midazolam^c^	32	35.7		
Feder et al. (2021)	RCT	Chronic PTSD	NR (assume racemic)	IV	0.5 mg/kg	3 doses/week	2 weeks	2 weeks	15 ketamine	87	39.3	PRISE	Weekly
									15 midazolam	67	38.5		
Fedgchin et al. (2019)	RCT	TRD (MDD)	Esketamine	Intranasal	56 or 84 mg	2 doses/week	4 weeks	4 weeks^a^	115 esketamine 56 mg + antidepressant	70	46.4	AEs	NR
									114 esketamine 84 mg + antidepressant	69	45.7		
									113 placebo + antidepressant	72	46.8		
Findeis et al. (2020) ^d^	Retrospective	MDD, BD, SZA	Esketamine	Subcutaneous or IV	0.25–0.5 mg/kg	3 doses/week	NR	NR	25 esketamine	60	49	Urinary erythrocyte, leukocyte, free haemoglobin, and protein concentration	A few times per week
Findeis et al. (2022) ^d^	Retrospective	MDD, BD, SZA	Esketamine	IV first dose, subsequently subcutaneously	0.25–0.5 mg/kg	Max. 3 doses/week	NR	NR	33 esketamine	53	47	Urinary erythrocytes and leukocytes	A few times per week
Gálvez et al. (2018)	RCT	TRD (MDD)	Racemic	Intranasal	100 mg	2 doses/week	4 weeks	4 weeks	3 ketamine	67	58.7	PRISE, BPIC-SS	NR
									2 midazolam	0	50.0		
George et al. (2017)	Crossover RCT, then OL	TRD (MDD and BD)	NR (assume racemic)	Subcutaneous	0.1, 0.2, 0.3, 0.4 or 0.5 mg/kg	1–2 doses/week	1–14 weeks	9–26 weeks	16 ketamine^e^	38	65.6	Urinary problem checklist	Twice weekly, then weekly
Glue et al. (2018)	OL one-arm	Treatment-refractory GAD and/or SAD	NR (assume racemic)	Subcutaneous	1 mg/kg	1–2 doses/week	14 weeks	14 weeks	20 ketamine	50	32	AEs	NR
Hartberg et al. (2018)	Retrospective	TRD (MDD)	NR (assume racemic)	Oral	0.5–7.0 mg/kg	2 doses/week – fortnightly	24–144 weeks	NR	37 ketamine	76	46^f^	AEs	NR
Ibrahim et al. (2012)	OL, then RCT^g^	TRD (MDD)	NR (assume racemic)	IV	0.5 mg/kg	1 dose	1 day	4 weeks	21 ketamine then placebo	38	47.2	AEs	During infusion and at 1 follow-up point
									21 ketamine then riluzole	38	47.2		
Lapidus et al. (2014)	Crossover RCT	MDD	Racemic	Intranasal	50 mg	1 dose	1 day	1 day^a^	20 ketamine^h^	50	48	SAFTEE	During infusion and at 1 follow-up point
									20 placebo^h^				
Loo et al. (2016)	Crossover RCT	TRD (MDD)	NR (assume racemic)	IV, intramuscular, or subcutaneous	Ascending dose: 0.1, 0.2, 0.3, 0.4, 0.5 mg/kg, plus 1 dose of midazolam	1 dose/week	1–6 weeks	4 h^a^	4 IV ketamine	50	52.8	SAFTEE	During infusion
									5 intramuscular ketamine	80	45.6		
									6 subcutaneous ketamine	83	48.2		
Loo et al. (2023)	RCT	TRD (MDD)	Racemic	Subcutaneous	Fixed dose (0.5 mg/kg) or flexible (0.5–0.9 mg/kg)	2 doses/week	4 weeks	4 weeks	35 fixed-dose ketamine	27	45.9	KSET	Twice per week
									38 fixed-dose midazolam	23	48.2		
									54 flexible-dose ketamine	14	44.5		
									54 fixed-dose midazolam	15	46.2		
Murrough et al. (2013a)	RCT	TRD (MDD)	NR (assume racemic)	IV	0.5 mg/kg	1 dose	1 day	1 week^a^	47 ketamine	55	46.9	PRISE	During infusion and at 1 follow-up point
									25 midazolam	44	42.7		
Murrough et al. (2013b)	OL one-arm	TRD (MDD)	Racemic	IV	0.5 mg/kg	3 doses/week	2 weeks	2 weeks^a^	24 ketamine	38	48.1	SAFTEE	During infusion and at 1 follow-up point
Ochs-Ross et al. (2020)	RCT	TRD (MDD)	Esketamine	Intranasal	28, 56 or 84 mg	2 doses/week	4 weeks	4 weeks	72 esketamine + antidepressant	63	70.6	AEs	NR
									65 placebo + antidepressant	62	69.4		
Sakurai et al. (2020)	Retrospective	TRD (MDD and BD)	NR (assume racemic)	IV	0.5 mg/kg	Acute: 2 doses/week, maintenance: 1 dose/2–6 weeks	At least 3 weeks, plus an open-ended maintenance phase	As long as treatment was ongoing, up to 44 weeks	87 ketamine	55	46	AEs	NR
Wajs et al. (2020)	OL one-arm	TRD (MDD)	Esketamine	Intranasal	28, 56 or 84 mg	Acute: 2 doses/week, maintenance: 1 dose/week or fortnightly	23 weeks,^f^ up to 52 weeks	52 weeks	802 ketamine + antidepressant	63	52.2	BPIC-SS, AEs	NR
Zaki et al. (2023)	OL one-arm	TRD (MDD)	Esketamine	Intranasal	28, 56 or 84 mg	NR	0–243 weeks, mean 137	Variable, as long as treatment was ongoing	1148 esketamine	67	49.6	AEs	NR

TRD: treatment-resistant depression; MDD: major depressive disorder; AEs: adverse events; NR: not reported; BD: bipolar disorder; IV: intravenous; OL: open label; PRISE: patient-related inventory of side effects; SAFTEE: systematic assessment of treatment emergent events; BPIC-SS: bladder pain/interstitial cystitis symptom score; KSET: ketamine side effect tool; RCT: randomised controlled trial; PTSD: post-traumatic stress disorder; SZA: schizoaffective disorder; GAD: general anxiety disorder; SAD: social anxiety disorder.

a An additional follow-up or trial phase was included, but AE information was not reported.

b Lithium or placebo was given in an RCT to responders to an initial dose of open-label ketamine, alongside three additional doses of ketamine.

c Demographic characteristics are presented for *N* participants randomised to ketamine or midazolam first. Safety information is presented for all participants receiving ketamine (*n* = 38) or midazolam (*n* = 31) on either infusion.

d Some crossover in patient samples.

e Urological data were available for *N* = 12 participants who entered the open-label phase.

f Median.

g Riluzole or placebo was given in an RCT after patients were given an initial dose of open-label ketamine.

h Crossover trial, demographic information provided for all 20 participants at baseline.

Regarding intervention characteristics, most studies (*n* = 13) only described formulation as ketamine (assumed to be racemic). The remainder specified racemic ketamine (*n* = 5), esketamine (*n* = 8) or compared esketamine to racemic ketamine (*n* = 1). Route of administration included IV (*n* = 9), intranasal (*n* = 8), oral (*n* = 3) or subcutaneous (*n* = 3) routes, two studies compared ketamine treatments (one including IV, intramuscular, and subcutaneous, and one including intranasal and IV) and two studies used a combination of subcutaneous and IV. Dosage in studies using intranasal routes ranged from 28 mg (d’Andrea et al., 2024; Daly et al., 2018; Ochs-Ross et al., 2020; Wajs et al., 2020; Zaki et al., 2023) to 100 mg (Gálvez et al., 2018). In studies using IV, intramuscular or subcutaneous routes, dosage ranged from 0.1 mg/kg (Fava et al., 2020; George et al., 2017; Loo et al., 2016) to 1.0 mg/kg (Fava et al., 2020; Glue et al., 2018), with the most common dose being 0.5 mg/kg. Oral ketamine dose was reported as mean 222 mg in one study (Al Shirawi et al., 2017), 0.5 mg/kg titrated up to 3.0 mg/kg max. in another (Can et al., 2021) and 0.5–7.0 mg/kg in the final study (Hartberg et al., 2018). Duration of treatment ranged from 1 day (Fava et al., 2020; Ibrahim et al., 2012; Lapidus et al., 2014; Murrough et al., 2013a) to 243 weeks (Zaki et al., 2023). Note that in many studies (especially in observational studies), dose, treatment schedule and duration were flexible and varied within patients based on response and tolerability. Twelve studies did not include a comparator treatment, seven included an active placebo (midazolam in all cases), five an inactive placebo and three studies compared ketamine treatments (differing either by route of administration or combination treatment). Most studies allowed stable concomitant medication (*n* = 15), five studies initiated a new antidepressant (SSRI or SNRI) alongside ketamine treatment, five excluded participants on psychotropic medication or antidepressants specifically and two did not report concomitant medication information.

Duration of follow-up of AE assessment ranged from 4 h (Loo et al., 2016) to 243 weeks (Zaki et al., 2023); however, some studies did not specify duration of follow-up (*n* = 5). Regarding urological assessments used, most studies did not specify the assessment used but did report urological AE data (*n* = 14), five studies used the Patient Related Inventory of Side Effects (PRISEs), four used the Systematic Assessment of Treatment Emergent Events, three used the Bladder Pain/Interstitial Cystitis Symptom Score (BPIC-SS), one used the urinary problem checklist and another used the Ketamine Side Effect Tool. Two studies assessed lab-based urinary parameters: urinary erythrocytes and leukocytes in one study and urinary erythrocyte, leukocyte, free haemoglobin and protein concentration in the other.

### Synthesis of results

#### Depressive disorders

##### Racemic ketamine

Five trials administered racemic IV ketamine for the treatment of unipolar TRD. A small open-label trial of six 0.5 mg/kg ketamine treatments over 2 weeks reported 2 (8%) patients at 240 min post-infusion and 1 (4.2%) patients at week 2 endorsing increased frequency of urination (Murrough et al., 2013b). An RCT comparing one 0.5 mg/kg dose of ketamine IV to active control (midazolam) found no cases of frequent, painful or difficulty urinating 240 min post-infusion in either arm (Murrough et al., 2013a). Similarly, an RCT of one 0.1, 0.2, 0.5 and 1.0 mg/kg ketamine or midazolam infusion found no cases of urological AEs in any arm (Fava et al., 2020). Two trials used 1 dose of ketamine IV open-label, then randomised participants to receive other treatments or placebo, either in combination with ketamine or alone. In the first trial, patients were randomised to riluzole or placebo for 4 weeks after one 0.5 mg/kg dose of IV ketamine (Ibrahim et al., 2012). At 230 min post-infusion, 1 (5%) patient reported increased frequency of urination in the ketamine then riluzole group, compared to 2 (11%) patients in the ketamine then placebo group. During the 4-week double-blind period, 1 (5%) patient reported increased frequency of urination in the ketamine then riluzole group, compared to 4 (21%) in the ketamine then placebo group. An additional 1 (5%) patient reported painful urination in the ketamine group than the placebo group. In the second study, patients who showed at least a partial response to an initial 0.5 mg/kg dose of IV ketamine were randomised to receive three further doses of ketamine plus lithium or placebo. Over the course of 4 weeks of treatment, 3 (16.6%) patients reported pollakiuria (more frequent urination) and 2 (11.1%) reported polyuria (increased volume of urine) in the ketamine plus lithium arm, compared to one, 1 (6.2%) patient reporting pollakiuria and none reporting polyuria in the ketamine plus placebo arm (Costi et al., 2019).

Two retrospective studies assessed 0.5 mg/kg IV ketamine maintenance treatment in patients with unipolar or bipolar TRD, for up to 44 and 49 weeks (Archer et al., 2018; Sakurai et al., 2020). No urinary side effects were reported in either study.

Two trials assessed the efficacy and safety of subcutaneous racemic ketamine for TRD. The first administered up to 5 doses of ketamine (0.1, 0.2, 0.3, 0.4 and 0.5 mg/kg) and 1 dose of midazolam randomly inserted in a crossover RCT phase, followed by a further 12 open-label ketamine doses for those who relapsed or did not achieve remission in the RCT phase (George et al., 2017). One (8.3%) patient reported occasional urological symptoms. A second RCT tested the safety and efficacy of either fixed- (0.5 mg/kg) or flexible-dose (0.5–0.9 mg/kg) ketamine compared with fixed- or flexible-dose midazolam over 4 weeks (Loo et al., 2023). Groups did not significantly differ in urological AEs: 4 (12.5%) patients in the fixed-dose ketamine arm, 6 (17.1%) in the fixed-dose midazolam arm, 13 (24.5%) in the flexible-dose ketamine arm and 9 (17.0%) in the flexible-dose midazolam arm reported urination AEs (frequency, pain, discomfort).

One trial examined the safety and feasibility of ascending doses of intramuscular, subcutaneous and IV racemic ketamine, with a midazolam control infusion randomly inserted (Loo et al., 2016). No urinary symptoms were reported in any treatment arm.

Three studies examined racemic oral ketamine. Two were retrospective studies, including data from patients with unipolar TRD receiving oral ketamine in the clinic. Al Shirawi et al. (2017) reported 1 (4.5%) case of lower urinary tract symptoms (polyuria and dysuria) in patients taking ketamine for at least 4 weeks (mean dose 222 mg), while Hartberg et al. (2018) reported no cases of bladder toxicity in patients taking 0.5 mg–7.0 mg/kg ketamine for 24–144 weeks. In an open-label trial of 6 weeks of 0.5 mg/kg oral ketamine in patients with MDD and chronic suicidality, Can et al. (2021) reported 1–2 (3.1%–6.3%) cases each of difficulty urinating and painful urination at each weekly follow-up.

Two RCTs tested the safety, feasibility, and efficacy of intranasal racemic ketamine for MDD or unipolar TRD. The first was a crossover trial, administering one 50 mg dose and a placebo a week apart in patients with MDD (Lapidus et al., 2014). 240 min post-ketamine, 0 cases of frequent urination were reported, compared to 1 (5%) post-placebo. One day post-treatment, 1 (5%) patient in each arm reported a frequent need to urinate. In the second study, patients with TRD received eight 100 mg doses of ketamine or midazolam over 4 weeks (Gálvez et al., 2018). No patients in either arm reported urinary symptoms on the PRISE, and mean BPIC-SS scores did not increase from baseline to post-treatment. However, the study was terminated after only five participants were enrolled due to acute problems with tolerability – motor incoordination resulted in participants being unable to self-administer the full dose of ketamine.

##### Esketamine

Six trials used esketamine nasal spray for the treatment of unipolar TRD. In a phase II trial, two (10%) patients treated with esketamine 56 mg reported polyuria over the 2-week treatment period, compared to none in the 28, 84 mg or placebo groups (Daly et al., 2018). An optional open-label phase and post-treatment follow-up phase were also included; however, AE data were not presented. Four larger phase III trials followed, the first reporting no cases of interstitial cystitis after at least 16 weeks of 56 or 84 mg esketamine treatment or placebo (Daly et al., 2019). In the second trial, which included patients with TRD who had shown a response to esketamine in previous short-term studies, 6 (5.2%) patients randomised to 56 mg esketamine and an oral antidepressant reported pollakiuria compared to 2 (1.7%) patients randomised to 84 mg plus an antidepressant, and 1 (0.9%) patient in the placebo plus antidepressant arm (Fedgchin et al., 2019). AE data were only reported for the 4-week double-blind phase, not the 24-week follow-up phase. In elderly patients with TRD, Ochs-Ross et al. (2020) reported 6 (8.3%) patients randomised to esketamine (28, 56 or 84 mg) and an oral antidepressant compared to 1 (1.5%) of patients randomised to placebo plus antidepressant contracted a urinary tract infection over the course of 4 weeks of treatment. In a large (*n* = 802), long-term, open-label trial, 136 (17.0%) patients reported treatment-emergent adverse events (TEAEs) related to renal or urinary disorders (Wajs et al., 2020). Of these, four patients had six serious TEAEs, none were deemed related to esketamine or led to discontinuation. Most cases of urinary tract symptoms were mild to moderate and resolved within 2 weeks. One patient discontinued due to urinary incontinence. A total of 14 patients (1.7%) had multiple episodes of BPIC-SS scores >18 (threshold for cystitis). Finally, a second large (*n* = 1148), long-term, ongoing open-label trial reported that 13.3% of patients developed UTIs during the trial, 2.7% dysuria, 2.4% pollakiuria, 1.3% micturition urgency, 1.3% nephrolithiasis, 1.0% haematuria and 1.0% urinary incontinence.

Two retrospective studies examined urinary toxicity markers throughout treatment (up to 47 doses) with subcutaneous or IV esketamine in patients with MDD, BD and schizoaffective disorder (Findeis et al., 2022; Findeis et al., 2020). No significant changes in urinary erythrocytes, leukocytes, free haemoglobin or protein concentration were found across time compared to baseline.

One retrospective cohort study compared the effects of IV racemic ketamine and intranasal esketamine, pooling data from two cohorts of patients with TRD (d’Andrea et al., 2024). No cases of uropathy were found over 4 weeks.

#### PTSD

Two small RCTs examined the effects of 0.5 mg/kg IV racemic ketamine compared with midazolam in patients with chronic PTSD. The first was a crossover trial administering 1 dose of ketamine and midazolam over the course of 2 weeks (Feder et al., 2014), finding that slightly more participants reported urinary symptoms on the PRISE (difficulty urinating, painful urination or frequent urination) during the infusion and 1 day post-treatment with midazolam (12.9% at both time points) than with ketamine (5.3% at both time points). The second study administered 6 infusions of ketamine or midazolam over 2 weeks (Feder et al., 2021). One patient (6.7%) in the ketamine arm reported urinary symptoms at week 1 follow-up compared to none in the midazolam arm, and two patients (13.4%) reported urinary symptoms in each arm at week 2.

#### Anxiety disorders

A one-arm open-label trial examined the safety and tolerability of 14 weeks of 1 mg/kg subcutaneous racemic ketamine maintenance treatment in patients with treatment-refractory GAD and/or SAD who were responders in an earlier ascending dose study (Glue et al., 2018). No patients reported symptoms of cystitis.

### Risk of bias

Of the randomised studies (*n* = 15), 1 (6.7%) was rated high risk of bias, 10 (66.7%) were rated some concerns and 4 (26.7%) were rated low risk of bias (see Supplemental material). Although all studies received a low risk of bias rating for bias due to deviations from the intended intervention and measurement of outcome, some studies raised concerns over the randomisation process, how missing data was dealt with and a lack of pre-registration of protocols. Of the non-randomised studies (*n* = 12), 10 (83.3%) were rated as serious risk of bias and two as moderate risk of bias (16.7%). Most studies received a low risk of bias for classification of interventions and deviations from intended interventions; however, many studies did not control for confounding factors, were unblinded and did not pre-register protocols.

## Discussion

This systematic review aimed to synthesise urological findings from trials and observational studies administering ketamine for the treatment of psychiatric disorders. Twenty-seven studies were included in total, including both real-world observational evidence and data from clinical trials. Urological symptoms were reported in 0%–24.5% of patients receiving ketamine treatment. Across studies, AEs were generally of mild to moderate severity. The most common symptoms reported across studies were frequent urination/pollakiuria (11 studies), dysuria (7 studies), polyuria, difficulty urinating and cystitis/UTI (3 studies each). Continuous outcome measures (urinary parameters and symptom questionnaires) did not show significant changes from baseline to follow-up.

In most randomised trials, rates of urological symptoms were equal to or lower than those reported in active or inactive control conditions (Daly et al., 2019; Fava et al., 2020; Feder et al., 2014, 2021; Gálvez et al., 2018; Lapidus et al., 2014; Loo et al., 2016; Murrough et al., 2013a). However, in three of the included intranasal esketamine trials, rates of urological symptoms were slightly elevated in those treated with esketamine compared to placebo (Daly et al., 2018; Fedgchin et al., 2019; Ochs-Ross et al., 2020). Additionally, in one study, urination AEs appeared to be more frequent in patients receiving flexible-dose racemic ketamine compared to fixed-dose ketamine and fixed- or flexible-dose midazolam (Loo et al., 2023). While these differences were not significant, a possible association between increased dose (in the flexible-dose arm) and increased frequency of AEs cannot be ruled out.

A number of points should be considered when interpreting the results of this review. Firstly, most studies did not include long-term assessment of urological symptoms or AEs, despite many including longer follow-ups or trial periods for documenting longer-term efficacy. Median duration of follow-up of urological symptoms included in this review was 4 weeks. In patients initiating ketamine treatment for the first time, urological symptoms are unlikely to develop over this timeframe. To our knowledge, no studies have assessed the relationship between duration of ketamine treatment and the development of urological symptoms. However, a case study of patients receiving ketamine for pain have reported symptoms developing after as little as 9 days (Grégoire et al., 2008), whilst others reported symptoms after 5 months (Storr and Quibell, 2009), albeit at higher doses than are used for treatment of depression and other psychiatric conditions. Five studies in our review assessed long-term (>6 months) ketamine treatment (all of which were retrospective or one-arm open-label trials in TRD). These studies reported urinary symptoms or AEs in 0%–17% of patients, most frequently urinary tract infections. From the limited data available, there is insufficient evidence to conclude whether long-term ketamine treatment for TRD is associated with serious urological symptoms, particularly in timeframes longer than 6 months.

Relatedly, many patients, especially in the United States, are now receiving ketamine in an unregulated fashion, outside of clinical trial protocols (Barnett et al., 2025). Esketamine nasal spray (Spravato) is monitored via an FDA Risk Evaluation and Mitigation Strategy (REMS), requiring it to be dispensed and administered under medical supervision. However, other ketamine products are not FDA approved for any indication and therefore are not part of a REMS programme. Despite this, ketamine is increasingly available for at-home use through telemedicine platforms, which market the drug for a wide variety of psychiatric disorders (FDA, 2023). Patients may be taking ketamine for long periods of time without adequate safety monitoring or reporting of urological and other AEs. While limited to published research studies, the findings of this review underscore an urgent need for improved clinical guidelines, tracking of urological symptoms, and education for prescribers and patients in early signs of KIUT.

That only 27 studies reported any urological AE assessment and were therefore eligible for inclusion in this review suggests a lack of systematic assessment of urological AEs in studies of ketamine treatment. A recent systematic review of the efficacy of ketamine treatment for psychiatric disorders included 50 randomised and non-randomised trials and retrospective studies (Walsh et al., 2022). Indeed, in a systematic review of side effects of ketamine treatment for depression, Short et al. (2018) found only five studies reporting urinary tract side effects. Notably, of the studies included in our review, 11 did not specify any systematic assessment of urological symptoms, reporting AE data only. Whilst passive monitoring of side effects is adequate for the detection of new, rare or serious side effects, active and structured enquiry may be required for known side effects (Short et al., 2018). Similarly, only two studies conducted lab-based urinary analyses, which may be more likely to pick up on subtle signs of early urothelial damage before symptoms emerge. In sum, the studies included in this review likely do not provide the full picture of urological side effects associated with ketamine treatment, due to both a lack of systematic assessment in included studies and a lack of any assessment at all in excluded studies.

### Limitations

A number of limitations should be noted. Only 15% of included studies were rated as low risk of bias. Many studies were unblinded and used subjective measures of symptoms (e.g. self-report), meaning that symptom reporting may have been influenced by the knowledge that patients were receiving ketamine. Additionally, self-report measures may be problematic in the context of ketamine, where the treatment itself may mask subjective symptoms of urological toxicity. Relatedly, urological symptoms are likely to arise only after significant urothelial damage has already occurred; therefore, self-report measures may miss early signs of toxicity.

Many studies did not include a control group, making it difficult to ascertain whether rates of bladder symptoms in those receiving ketamine were any higher than those reported with no treatment or a comparator. Bias may have also arisen in several studies in the participant selection process, whereby some studies only included participants who had responded to ketamine treatment before entering the study (therefore excluding participants who may have dropped out due to side effects).

Included studies almost exclusively investigated ketamine treatment for depressive disorders, which is not surprising given the stage of development for this indication compared to other psychiatric disorders. Nonetheless, studies have administered repeated ketamine treatments for other indications, including alcohol and substance use disorders (Grabski et al., 2022; Krupitsky et al., 2007) and eating disorders (Mills et al., 1998). Monitoring of urinary AEs may be more pertinent in these patients who often present with physical complications due to their illness (AshaRani et al., 2023; Puckett, 2023).

## Conclusion

There is limited data available assessing urological side effects of ketamine treatment, especially over the long term. This represents a major gap in the literature, given the known risks of ketamine-associated urological toxicity. Rates of urological symptoms were generally low and mild to moderate in severity; however, confidence in these results is limited by methodological limitations, including a lack of systematic assessments and short follow-up periods. Future studies examining ketamine’s effect in clinical populations should include a systematic evaluation of urinary tract symptoms. This could be achieved through the use of rating scales, such as the BPIC-SS. Furthermore, pre-post measurements of urinary markers offer a cheap and non-invasive option for pre-symptomatic detection. Such objective markers may be of particular importance given the potential confounding analgesic effect of ketamine. Inclusion of such measures should not be optional but mandated as part of the study design. Examining the relationship between early urinary markers and any subsequent urinary symptom development may help enhance safety protocols for clinical use. A proactive strategy is particularly important among clinical populations with serious mental illness, given the relationship with chronic physical health conditions (Pizzol et al., 2023). Implementing these measurements would improve the accuracy and completeness of safety monitoring and our understanding of this potentially serious adverse effect.

## Supplemental Material

sj-docx-1-jop-10.1177_02698811251350267 – Supplemental material for Urological symptoms following ketamine treatment for psychiatric disorders: A systematic review Supplemental material, sj-docx-1-jop-10.1177_02698811251350267 for Urological symptoms following ketamine treatment for psychiatric disorders: A systematic review by Jess Kerr-Gaffney, Anna Tröger, Alice Caulfield, Philipp Ritter, James Rucker and Allan H Young in Journal of Psychopharmacology

## References

[bibr1-02698811251350267] Al Shirawi MI Kennedy SH Ho KT , et al. (2017) Oral ketamine in treatment-resistant depression. J Clin Psychopharmacol 37: 464–467. 28514237 10.1097/JCP.0000000000000717 PMC5491240

[bibr2-02698811251350267] Albott CS Lim KO Forbes MK , et al. (2018) Efficacy, safety, and durability of repeated ketamine infusions for comorbid posttraumatic stress disorder and treatment-resistant depression. J Clin Psychiatry 79: 17m11634. 10.4088/JCP.17m11634 29727073

[bibr3-02698811251350267] Anderson DJ Zhou J Cao D , et al. (2022) Ketamine-induced cystitis: A comprehensive review of the urologic effects of this psychoactive drug. Health Psychol Res 10: 38247. 36118982 10.52965/001c.38247 PMC9476224

[bibr4-02698811251350267] Andrade C (2017) Ketamine for depression, 4: In what dose, at what rate, by what route, for how long, and at what frequency? J Clin Psychiatry 78: e852–e857. 10.4088/JCP.17f11738 28749092

[bibr5-02698811251350267] Archer S Chrenek C Swainson J (2018) Maintenance ketamine therapy for treatment-resistant depression. J Clin Psychopharmacol 38: 380–384. 29912788 10.1097/JCP.0000000000000894

[bibr6-02698811251350267] AshaRani PV Karuvetil MZ Brian TYW , et al. (2023) Prevalence and correlates of physical comorbidities in alcohol use disorder (AUD): A pilot study in treatment-seeking population. Int J Mental Health Addict 21: 2508–2525. 10.1007/s11469-021-00734-5 PMC8783789 35095353

[bibr7-02698811251350267] atai Life Sciences (2023) atai Life Sciences Announces Results from Phase 2a Trial of PCN-101 (R-ketamine) for Treatment-Resistant Depression. Available at: https://atai.life/2023/01/09/atai-life-sciences-announces-results-from-phase-2a-trial-of-pcn-101-r-ketamine-for-treatment-resistant-depression/ (accessed 13 January 2025).

[bibr8-02698811251350267] Aziz W Babb C Brown D , et al. (2017) Ketamine role in schizoaffective disorder depressive type. J Addict Res Ther 8: 346.

[bibr9-02698811251350267] Bahji A Vazquez GH Zarate CA (2021) Comparative efficacy of racemic ketamine and esketamine for depression: A systematic review and meta-analysis. J Affect Disord 278: 542–555. 33022440 10.1016/j.jad.2020.09.071 PMC7704936

[bibr10-02698811251350267] Barnett BS Weiss RD Sanacora G (2025) Strengthening safeguards for psychiatric uses of ketamine. JAMA Psychiatry 82: 333–334. 39937465 10.1001/jamapsychiatry.2024.4787

[bibr11-02698811251350267] Berman RM Cappiello A Anand A , et al. (2000) Antidepressant effects of ketamine in depressed patients. Biol Psychiatry 47: 351–354. 10.1016/S0006-3223(99)00230-9 10686270

[bibr12-02698811251350267] Bloch MH Wasylink S Landeros-Weisenberger A , et al. (2012) Effects of Ketamine in treatment-refractory obsessive-compulsive disorder. Biol Psychiatry 72: 964–970. 22784486 10.1016/j.biopsych.2012.05.028 PMC3667652

[bibr13-02698811251350267] Can AT Hermens DF Dutton M , et al. (2021) Low dose oral ketamine treatment in chronic suicidality: An open-label pilot study. Transl Psychiatry 11: 101. 33542187 10.1038/s41398-021-01230-z PMC7862447

[bibr14-02698811251350267] Costi S Soleimani L Glasgow A , et al. (2019) Lithium continuation therapy following ketamine in patients with treatment resistant unipolar depression: A randomized controlled trial. Neuropsychopharmacology 44: 1812–1819. 30858518 10.1038/s41386-019-0365-0 PMC6784998

[bibr15-02698811251350267] d’Andrea G Pettorruso M Di Lorenzo G , et al. (2024) The rapid antidepressant effectiveness of repeated dose of intravenous ketamine and intranasal esketamine: A post-hoc analysis of pooled real-world data. J Affect Disord 348: 314–322. 38145840 10.1016/j.jad.2023.12.038

[bibr16-02698811251350267] Daly EJ Singh JB Fedgchin M , et al. (2018) Efficacy and safety of intranasal esketamine adjunctive to oral antidepressant therapy in treatment-resistant depression: A randomized clinical trial. JAMA Psychiatry 75: 139–148. 29282469 10.1001/jamapsychiatry.2017.3739 PMC5838571

[bibr17-02698811251350267] Daly EJ Trivedi MH Janik A , et al. (2019) Efficacy of esketamine nasal spray plus oral antidepressant treatment for relapse prevention in patients with treatment-resistant depression. JAMA Psychiatry 76: 893. 31166571 10.1001/jamapsychiatry.2019.1189 PMC6551577

[bibr18-02698811251350267] Dean RL Hurducas C Hawton K , et al. (2021) Ketamine and other glutamate receptor modulators for depression in adults with unipolar major depressive disorder. Cochrane Database Syst Rev 9: CD011612. 10.1002/14651858.CD011612.pub3 PMC8434915 34510411

[bibr19-02698811251350267] Domino EF (1980) History and pharmacology of PCP and PCP-related analogs. J Psyched Drugs 12: 223–227. 10.1080/02791072.1980.10471430 7431418

[bibr20-02698811251350267] Ebert B Mikkelsen S Thorkildsen C , et al. (1997) Norketamine, the main metabolite of ketamine, is a non-competitive NMDA receptor antagonist in the rat cortex and spinal cord. Eur J Pharmacol 333: 99–104. 9311667 10.1016/s0014-2999(97)01116-3

[bibr21-02698811251350267] Fava M Freeman MP Flynn M , et al. (2020) Double-blind, placebo-controlled, dose-ranging trial of intravenous ketamine as adjunctive therapy in treatment-resistant depression (TRD). Mol Psychiatry 25: 1592–1603. 30283029 10.1038/s41380-018-0256-5 PMC6447473

[bibr22-02698811251350267] Feder A Costi S Rutter SB , et al. (2021) A randomized controlled trial of repeated ketamine administration for chronic posttraumatic stress disorder. Am J Psychiatry 178: 193–202. 33397139 10.1176/appi.ajp.2020.20050596

[bibr23-02698811251350267] Feder A Parides MK Murrough JW , et al. (2014) Efficacy of intravenous ketamine for treatment of chronic posttraumatic stress disorder: A randomized clinical trial. JAMA Psychiatry 71: 681–688. 24740528 10.1001/jamapsychiatry.2014.62

[bibr24-02698811251350267] Fedgchin M Trivedi M Daly EJ , et al. (2019) Efficacy and safety of fixed-dose esketamine nasal spray combined with a new oral antidepressant in treatment-resistant depression: Results of a randomized, double-blind, active-controlled study (TRANSFORM-1). Int J Neuropsychopharmacol 22: 616–630. 31290965 10.1093/ijnp/pyz039 PMC6822141

[bibr25-02698811251350267] Findeis H Ludwig V Mikolas P , et al. (2022) Practical aspects of ketamine treatment—Safety, combination treatment and comorbidities. Nervenarzt 93: 243–253. 35171310 10.1007/s00115-021-01260-4

[bibr26-02698811251350267] Findeis H Sauer C Cleare A , et al. (2020) Urothelial toxicity of esketamine in the treatment of depression. Psychopharmacology 237: 3295–3302. 32712681 10.1007/s00213-020-05611-y PMC7561544

[bibr27-02698811251350267] Food and Drug Administration (2023) FDA warns patients and health care providers about potential risks associated with compounded ketamine products, including oral formulations, for the treatment of psychiatric disorders. Available at: https://www.fda.gov/drugs/human-drug-compounding/fda-warns-patients-and-health-care-providers-about-potential-risks-associated-compounded-ketamine (accessed 20 April 2025).

[bibr28-02698811251350267] Gálvez V Li A Huggins C , et al. (2018) Repeated intranasal ketamine for treatment-resistant depression – the way to go? Results from a pilot randomised controlled trial. J Psychopharmacol 32: 397–407. 29542371 10.1177/0269881118760660

[bibr29-02698811251350267] George D Gálvez V Martin D , et al. (2017) Pilot randomized controlled trial of titrated subcutaneous ketamine in older patients with treatment-resistant depression. Am J Geriatr Psychiatry 25: 1199–1209. 28739263 10.1016/j.jagp.2017.06.007

[bibr30-02698811251350267] Glue P Neehoff SM Medlicott NJ , et al. (2018) Safety and efficacy of maintenance ketamine treatment in patients with treatment-refractory generalised anxiety and social anxiety disorders. J Psychopharmacol 32: 663–667. 29561204 10.1177/0269881118762073

[bibr31-02698811251350267] Grabski M McAndrew A Lawn W , et al. (2022) Adjunctive ketamine with relapse prevention–based psychological therapy in the treatment of alcohol use disorder. Am J Psychiatry 179: 152–162. 35012326 10.1176/appi.ajp.2021.21030277

[bibr32-02698811251350267] Grégoire MC MacLellan DL Finley GA (2008) A pediatric case of ketamine-associated cystitis. Urology 71: 1232–1233. 10.1016/j.urology.2007.11.141 18455768

[bibr33-02698811251350267] Hartberg J Garrett-Walcott S De Gioannis A (2018) Impact of oral ketamine augmentation on hospital admissions in treatment-resistant depression and PTSD: A retrospective study. Psychopharmacology 235: 393–398. 29151192 10.1007/s00213-017-4786-3

[bibr34-02698811251350267] Hashimoto K (2020) Molecular mechanisms of the rapid-acting and long-lasting antidepressant actions of (R)-ketamine. Biochem Pharmacol 177: 113935. 32224141 10.1016/j.bcp.2020.113935

[bibr35-02698811251350267] Ibrahim L Diazgranados N Franco-Chaves J , et al. (2012) Course of improvement in depressive symptoms to a single intravenous infusion of ketamine vs add-on riluzole: Results from a 4-week, double-blind, placebo-controlled study. Neuropsychopharmacology 37: 1526–1533. 22298121 10.1038/npp.2011.338 PMC3327857

[bibr36-02698811251350267] Khorramzadeh E Lotfy AO (1973) The use of ketamine in psychiatry. Psychosomatics 14: 344–346. 4800188 10.1016/S0033-3182(73)71306-2

[bibr37-02698811251350267] Krupitsky EM Grinenko AY (1997) Ketamine psychedelic therapy (KPT): A review of the results of ten years of research. J Psychoact Drugs 29: 165–183. 10.1080/02791072.1997.10400185 9250944

[bibr38-02698811251350267] Krupitsky EM Burakov AM Dunaevsky IV , et al. (2007) Single versus repeated sessions of ketamine-assisted psychotherapy for people with heroin dependence. J Psychoact Drugs 39: 13–19. 10.1080/02791072.2007.10399860 17523581

[bibr39-02698811251350267] Lapidus KAB Levitch CF Perez AM , et al. (2014) A randomized controlled trial of intranasal ketamine in major depressive disorder. Biol Psychiatry 76: 970–976. 24821196 10.1016/j.biopsych.2014.03.026 PMC4185009

[bibr40-02698811251350267] Leal GC Souza-Marques B Mello RP , et al. (2023) Arketamine as adjunctive therapy for treatment-resistant depression: A placebo-controlled pilot study. J Affect Disord 330: 7–15. 36871913 10.1016/j.jad.2023.02.151

[bibr41-02698811251350267] Loo C Glozier N Barton D , et al. (2023) Efficacy and safety of a 4-week course of repeated subcutaneous ketamine injections for treatment-resistant depression (KADS study): Randomised double-blind active-controlled trial. Br J Psychiatry 223: 533–541. 38108319 10.1192/bjp.2023.79 PMC10727911

[bibr42-02698811251350267] Loo C Gálvez V O’Keefe E , et al. (2016) Placebo-controlled pilot trial testing dose titration and intravenous, intramuscular and subcutaneous routes for ketamine in depression. Acta Psychiatr Scand 134: 48–56. 27028832 10.1111/acps.12572

[bibr43-02698811251350267] Mills I Park G Manara A , et al. (1998) Treatment of compulsive behaviour in eating disorders with intermittent ketamine infusions. QJM 91: 493–503. 9797933 10.1093/qjmed/91.7.493

[bibr44-02698811251350267] Morgan CJA Curran HV (2006) Acute and chronic effects of ketamine upon human memory: A review. Psychopharmacology 188: 408–424. 17006715 10.1007/s00213-006-0572-3

[bibr45-02698811251350267] Morgan CJA Curran HV (2012) Ketamine use: A review. Addiction 107: 27–38. 21777321 10.1111/j.1360-0443.2011.03576.x

[bibr46-02698811251350267] Morrison RL Singh J Daly E , et al. (2024) Effect of esketamine nasal spray on cognition in patients with treatment-resistant depression: Results from four phase 3 studies. Int J Neuropsychopharmacol 27: pyae046. 10.1093/ijnp/pyae046 PMC11561565 39514643

[bibr47-02698811251350267] Muetzelfeldt L Kamboj SK Rees H , et al. (2008) Journey through the K-hole: Phenomenological aspects of ketamine use. Drug Alcohol Depend 95: 219–229. 18355990 10.1016/j.drugalcdep.2008.01.024

[bibr48-02698811251350267] Murrough JW Iosifescu DV Chang LC , et al. (2013a) Antidepressant efficacy of ketamine in treatment-resistant major depression: A two-site randomized controlled trial. Am J Psychiatry 170: 1134–1142. 23982301 10.1176/appi.ajp.2013.13030392 PMC3992936

[bibr49-02698811251350267] Murrough JW Perez AM Pillemer S , et al. (2013b) Rapid and longer-term antidepressant effects of repeated ketamine infusions in treatment-resistant major depression. Biol Psychiatry 74: 250–256. 22840761 10.1016/j.biopsych.2012.06.022 PMC3725185

[bibr50-02698811251350267] Ng J Lui LMW Rosenblat JD , et al. (2021) Ketamine-induced urological toxicity: Potential mechanisms and translation for adults with mood disorders receiving ketamine treatment. Psychopharmacology 238: 917–926. 33484298 10.1007/s00213-021-05767-1

[bibr51-02698811251350267] Ochs-Ross R Daly EJ Zhang Y , et al. (2020) Efficacy and safety of esketamine nasal spray plus an oral antidepressant in elderly patients with treatment-resistant depression—TRANSFORM-3. Am J Geriatr Psychiatry 28: 121–141. 31734084 10.1016/j.jagp.2019.10.008

[bibr52-02698811251350267] Page MJ Moher D Bossuyt PM , et al. (2021) PRISMA 2020 explanation and elaboration: Updated guidance and exemplars for reporting systematic reviews. BMJ; 372: n160. 10.1136/bmj.n160 PMC8005925 33781993

[bibr53-02698811251350267] Pizzol D Trott M Butler L , et al. (2023) Relationship between severe mental illness and physical multimorbidity: A meta-analysis and call for action. BMJ Mental Health 26: e300870. 10.1136/bmjment-2023-300870 PMC10619039 37907331

[bibr54-02698811251350267] Popova V Daly EJ Trivedi M , et al. (2019) Efficacy and safety of flexibly dosed esketamine nasal spray combined with a newly initiated oral antidepressant in treatment-resistant depression: A randomized double-blind active-controlled study. Am J Psychiatry 176: 428–438. 31109201 10.1176/appi.ajp.2019.19020172

[bibr55-02698811251350267] Puckett L (2023) Renal and electrolyte complications in eating disorders: A comprehensive review. J Eat Disord 11: 26. 36803805 10.1186/s40337-023-00751-w PMC9942359

[bibr56-02698811251350267] Reif A Bitter I Buyze J , et al. (2023) Esketamine nasal spray versus quetiapine for treatment-resistant depression. N Engl J Med 389: 1298–1309. 37792613 10.1056/NEJMoa2304145

[bibr57-02698811251350267] Romeo B Choucha W Fossati P , et al. (2015) Meta-analysis of short- and mid-term efficacy of ketamine in unipolar and bipolar depression. Psychiatry Res 230: 682–688. 26548981 10.1016/j.psychres.2015.10.032

[bibr58-02698811251350267] Sakurai H Jain F Foster S , et al. (2020) Long-term outcome in outpatients with depression treated with acute and maintenance intravenous ketamine: A retrospective chart review. J Affect Disord 276: 660–666. 32871698 10.1016/j.jad.2020.07.089

[bibr59-02698811251350267] Short B Fong J Galvez V , et al. (2018) Side-effects associated with ketamine use in depression: A systematic review. Lancet Psychiatry 5: 65–78. 28757132 10.1016/S2215-0366(17)30272-9

[bibr60-02698811251350267] Sterne JA Hernán MA Reeves BC , et al. (2016) ROBINS-I: A tool for assessing risk of bias in non-randomised studies of interventions. BMJ; 355: i4919. 10.1136/bmj.i4919 PMC5062054 27733354

[bibr61-02698811251350267] Sterne JA Savovic J Page M , et al. (2019) RoB 2: A revised tool for assessing risk of bias in randomised trials. BMJ 366: l4898. 10.1136/bmj.l4898 31462531

[bibr62-02698811251350267] Storr T Quibell R (2009) Can ketamine prescribed for pain cause damage to the urinary tract? Palliat Med 23: 670–672. 19648225 10.1177/0269216309106828

[bibr63-02698811251350267] Wajs E Aluisio L Holder R , et al. (2020) Esketamine nasal spray plus oral antidepressant in patients with treatment-resistant depression. J Clin Psychiatry 81: 19m12891. 10.4088/JCP.19m12891 32316080

[bibr64-02698811251350267] Walsh Z Mollaahmetoglu OM Rootman J , et al. (2022) Ketamine for the treatment of mental health and substance use disorders: Comprehensive systematic review. BJPsych Open 8: 1–12. 35040425 10.1192/bjo.2022.5 PMC8811778

[bibr65-02698811251350267] Weiner AL Vieira L McKay CA , et al. (2000) Ketamine abusers presenting to the Emergency Department: A case series. J Emerg Med 18: 447–451. 10802423 10.1016/s0736-4679(00)00162-1

[bibr66-02698811251350267] Winstock AR Mitcheson L Gillatt DA , et al. (2012) The prevalence and natural history of urinary symptoms among recreational ketamine users. BJU Int 110: 1762–1766. 22416998 10.1111/j.1464-410X.2012.11028.x

[bibr67-02698811251350267] Wood D Cottrell A Baker SC , et al. (2011) Recreational ketamine: From pleasure to pain. BJU Int 107: 1881–1884. 21314885 10.1111/j.1464-410X.2010.10031.x

[bibr68-02698811251350267] Zaki N Chen L Lane R , et al. (2023) Long-term safety and maintenance of response with esketamine nasal spray in participants with treatment-resistant depression: Interim results of the SUSTAIN-3 study. Neuropsychopharmacology 48: 1225–1233. 37173512 10.1038/s41386-023-01577-5 PMC10267177

[bibr69-02698811251350267] Zanos P Moaddel R Morris PJ , et al. (2018) Ketamine and ketamine metabolite pharmacology: Insights into therapeutic mechanisms. Pharmacol Rev 70: 621–660. 29945898 10.1124/pr.117.015198 PMC6020109

