# Supplementary material for: Urological symptoms following ketamine treatment for psychiatric disorders: A systematic review
Source: J Psychopharmacol. 2025 Jun 30;39(10):1103–13. doi: 10.1177/02698811251350267 (PMC12572349; doi:10.1177/02698811251350267)
Supplement: sj-docx-1-jop-10.1177_02698811251350267 – Supplemental material for Urological symptoms following ketamine treatment for psychiatric disorders: A systematic review [file sj-docx-1-jop-10.1177_02698811251350267.docx]

**Supplementary material**


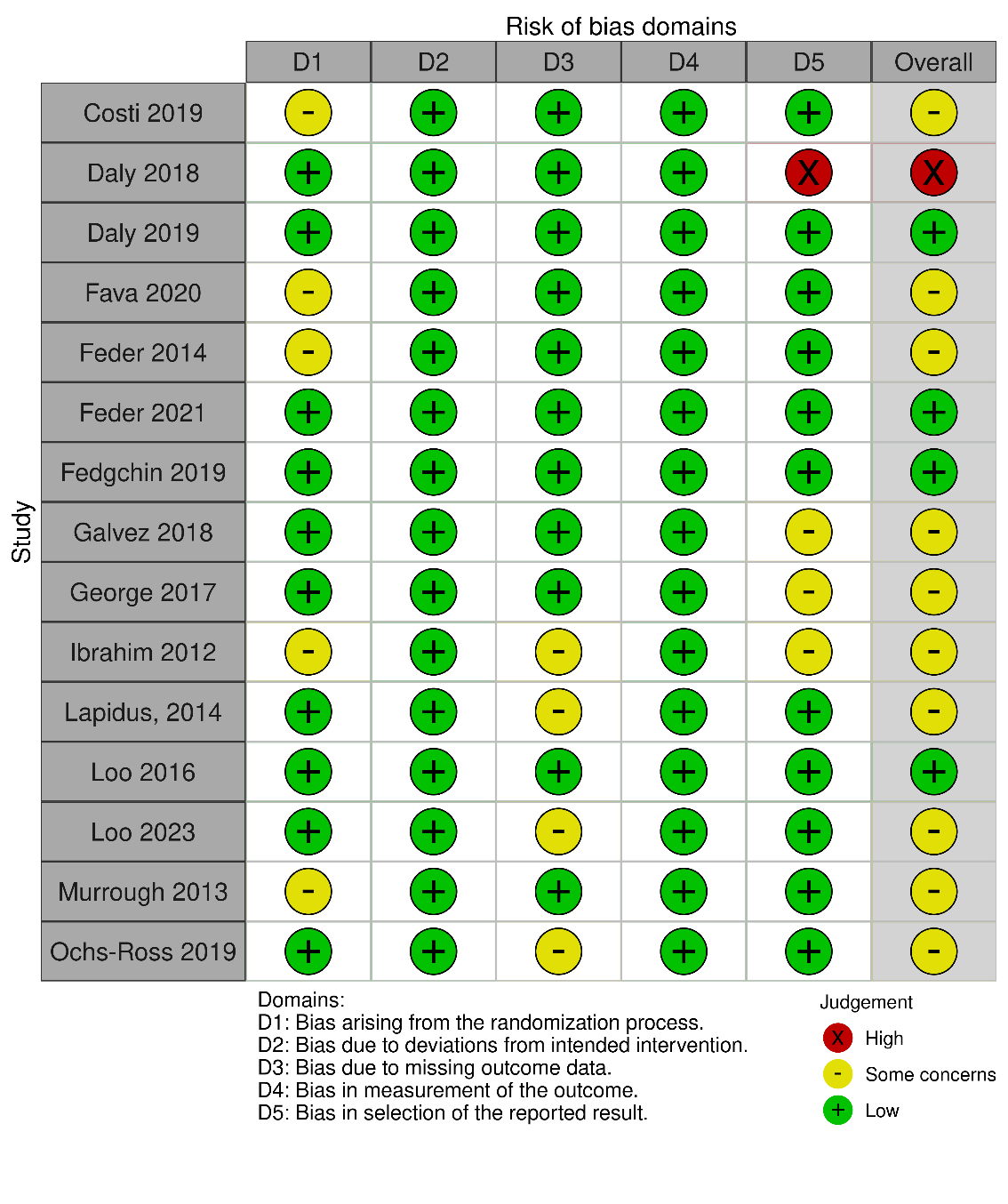


Figure S1. Risk of bias ratings for randomised studies.


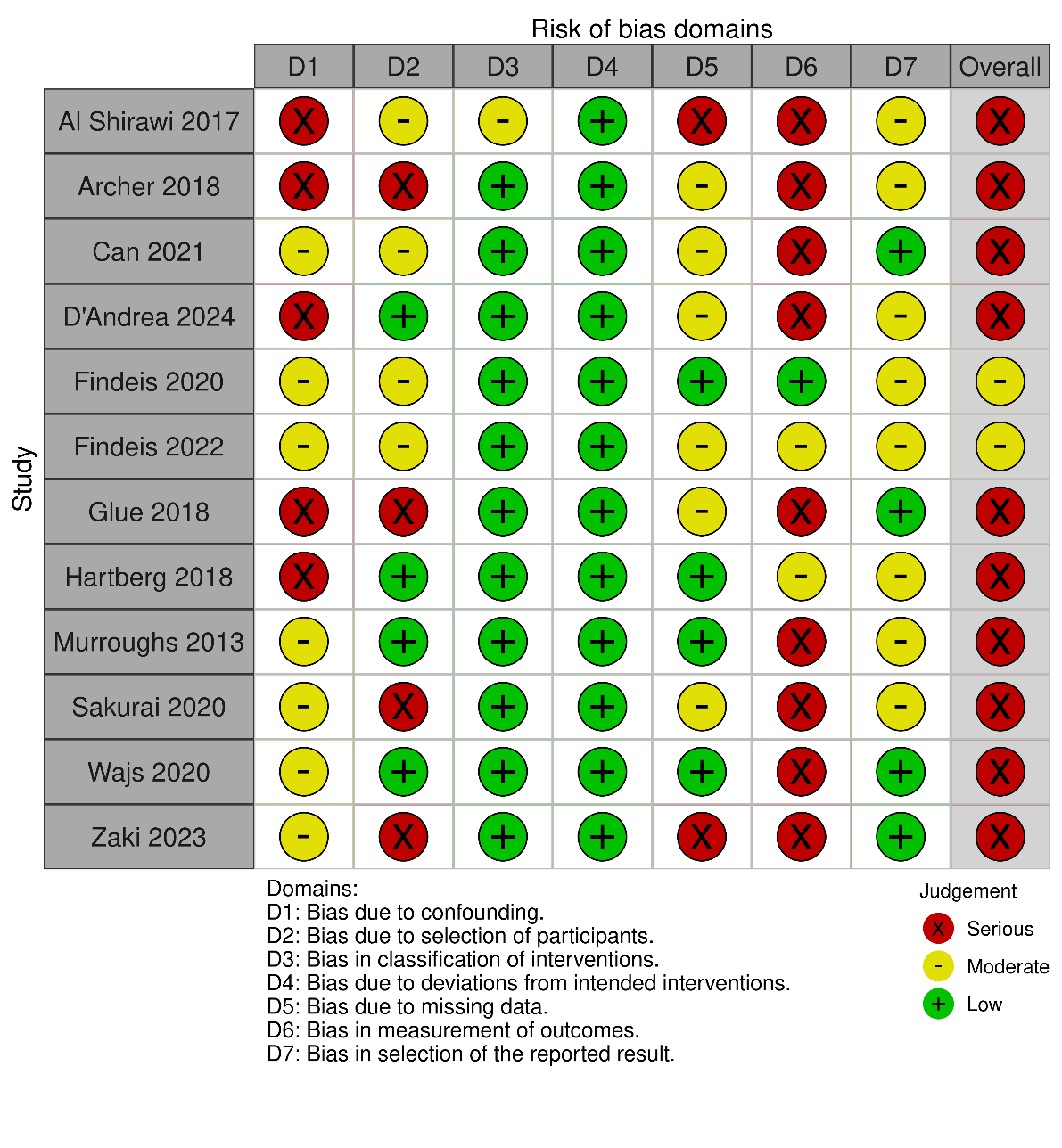


Figure 2. Risk of bias ratings for non-randomised studies.
